# Comprehensive Analysis of DWARF14-LIKE2 (DLK2) Reveals Its Functional Divergence from Strigolactone-Related Paralogs

**DOI:** 10.3389/fpls.2017.01641

**Published:** 2017-09-22

**Authors:** Attila Végh, Norbert Incze, Attila Fábián, Heqiang Huo, Kent J. Bradford, Ervin Balázs, Vilmos Soós

**Affiliations:** ^1^Department of Applied Genomics, Agricultural Institute, Centre for Agricultural Research, Hungarian Academy of Sciences Martonvasar, Hungary; ^2^Department of Plant Cell Biology, Agricultural Institute, Centre for Agricultural Research, Hungarian Academy of Sciences Martonvasar, Hungary; ^3^Department of Plant Sciences, Seed Biotechnology Center, University of California, Davis, Davis CA, United States

**Keywords:** AtD14, butenolide, DLK2, KAI2, light, MAX2, strigolactone

## Abstract

Strigolactones (SLs) and related butenolides, originally identified as active seed germination stimulants of parasitic weeds, play important roles in many aspects of plant development. Two members of the D14 α/β hydrolase protein family, DWARF14 (D14) and KARRIKIN INSENSITIVE2 (KAI2) are essential for SL/butenolide signaling. The third member of the family in Arabidopsis, DWARF 14-LIKE2 (DLK2) is structurally very similar to D14 and KAI2, but its function is unknown. We demonstrated that DLK2 does not bind nor hydrolyze natural (+)5-deoxystrigol [(+)5DS], and weakly hydrolyzes non-natural strigolactone (-)5DS. A detailed genetic analysis revealed that DLK2 does not affect SL responses and can regulate seedling photomorphogenesis. *DLK2* is upregulated in the dark dependent upon KAI2 and PHYTOCHROME INTERACTING FACTORS (PIFs), indicating that DLK2 might function in light signaling pathways. In addition, unlike its paralog proteins, DLK2 is not subject to *rac*-GR24-induced degradation, suggesting that DLK2 acts independently of MORE AXILLARY GROWTH2 (MAX2); however, regulation of DLK2 transcription is mostly accomplished through MAX2. In conclusion, these data suggest that DLK2 represents a divergent member of the DWARF14 family.

## Introduction

Butenolides are lactone-containing heterocyclic molecules with important biochemical and physiological roles in plant life. Although previously recognized as secondary metabolites, some types of butenolides were recently classified as plant hormones (Gomez-Roldan et al., 2008; Umehara et al., 2008). Strigolactones (SLs) are carotenoid-derived molecules bearing essential butenolide moieties that were originally described as chemical cues promoting seed germination of parasitic *Striga* species (Cook et al., 1966; reviewed in Al-Babili and Bouwmeester, 2015). It has since become evident that SLs are involved in controlling a wide range of plant developmental processes, including root architecture, establishment of mycorrhiza, stature and shoot branching, seedling growth, senescence, leaf morphology and cambial activity (Snowden et al., 2005; Besserer et al., 2006; Gomez-Roldan et al., 2008; Umehara et al., 2008; Agusti et al., 2011; Kapulnik et al., 2011; Waters et al., 2012b; Ueda and Kusaba, 2015; Soundappan et al., 2015). SLs are synthesized via a sequential cleavage of all-*trans*-*β*-carotene by DWARF27 (Waters et al., 2012a) and the resulting 9-*cis-β*-carotene by MORE AXILLARY GROWTH3 and 4 (MAX3,4; Alder et al., 2008). The SL precursor carlactone is then transported through the xylem and biologically active SLs are formed by MAX1 and its homologs (Seto et al., 2014; Zhang Y. et al., 2014; Al-Babili and Bouwmeester, 2015) and LATERAL BRANCHING OXIDOREDUCTASE (LBO; Brewer et al., 2016). Cumulative evidence supports the idea that the DWARF14 α/β-fold hydrolase (D14) functions as a SL receptor and is required for the perception of the SL signal in *Petunia* (DECREASED APICAL DOMINANCE2, DAD2; Hamiaux et al., 2012), rice (*Oryza sativa*) (D14; Arite et al., 2009), Arabidopsis (AtD14; Waters et al., 2012b) and pea (RAMOSUS3, RMS3; de Saint Germain et al., 2016). Upon binding, D14 proteins hydrolyze SL by action of its conserved Ser-His-Asp catalytic triad, followed by thermal destabilization of the proteins (Hamiaux et al., 2012; Yao et al., 2016). As a consequence, the structural rearrangement of D14 proteins in the presence of SL enables the protein to physically interact with the F-box proteins MAX2 (D3 in rice) and SMAX1-LIKE (SMXL) (D53 in rice; Zhou et al., 2013) family proteins SMXL 6, 7 and 8 (Wang et al., 2015; Soundappan et al., 2015) to form a Skp-Cullin-F-box (SCF) ubiquitin ligase complex that polyubiquitinates SMXLs and targets them for degradation by the 26S proteasome. The subsequent signaling events are largely unknown, but tentatively the mechanism is similar to other systems employing targeted protein degradation (Smith and Li, 2014; Wallner et al., 2016).

In Arabidopsis, two paralogs of AtD14 have been identified (Waters et al., 2012b). One paralog, KARRIKIN INSENSITIVE2 (KAI2) was identified in a mutant in L*er* background (Waters et al., 2012b) which showed insensitivity to karrikin (KAR), a butenolide-type germination stimulant from smoke water (Flematti et al., 2004; Nelson et al., 2010). Although both AtD14 and KAI2 signaling pathways converge upon MAX2 and might employ similar mechanisms to transduce the signal, the two proteins regulate separate physiological events. Unlike AtD14, KAI2 genetically interacts with the other members of the SMXL family (SMAX1 and SMXL2), which redundantly regulate SL- and KAR-related gene expression (Stanga et al., 2013, 2016). KAI2 is required in Arabidopsis primarily for seed germination, normal seedling photomorphogenic responses, and leaf development (Waters et al., 2012b, 2015b; Soundappan et al., 2015; Bennett et al., 2016), while in rice KAI2 is essential to the perception of symbiotic signal needed for mycorrhizal association (Gutjahr et al., 2015). This functional divergence suggested that KAI2 is a component of an SL-independent signaling pathway that perceives a hypothetical butenolide ligand, termed KL (for KAI2 ligand; Conn and Nelson, 2016; Morffy et al., 2016), which is neither SL nor karrikin. Evidence supporting this hypothesis is that AtD14 shows high affinity toward both (-)5DS and the natural (+)5DS signal, while KAI2 stereospecifically binds and hydrolyzes only the non-natural (-)5DS SL (Scaffidi et al., 2014; Waters et al., 2015b). Comparisons of mutants from different ecotypes of Arabidopsis led to the isolation of a loss-of-function allele of *KAI2* in Col-0 designated as *htl-3* (Toh et al., 2014).

Very little is known about DWARF14-LIKE2 (DLK2), the third member of the DWARF14 protein family, to which no physiological role has been assigned as yet. Arabidopsis *dlk2* mutants in Col-0 background exhibit normal seed dormancy, photomorphogenic responses, and branching phenotypes (Waters et al., 2012b), although in rice DLK2 may regulate mesocotyl elongation in the dark (Kameoka and Kyozuka, 2015). *DLK2* gene expression was recognized as an excellent marker for SL or KAR action (Waters et al., 2012b; Sun et al., 2016), and as a karrikin-responsive transcript in germinating lettuce (*Lactuca sativa*) achenes (Soós et al., 2012). *DLK2* is upregulated through the action of AtD14 or KAI2 in seedlings after SL or karrikin treatments, and its normal expression is highly dependent on MAX2 and KAI2 (Waters and Smith, 2013; Stanga et al., 2013; Scaffidi et al., 2014). Interestingly*, smxl1,2* double mutants exhibit increased *DLK2* expression, indicating that KAR/KL signaling is constitutively activated in these mutants (Stanga et al., 2016). This butenolide-dependent expression has been hypothesized to be a negative feedback system in which DLK2 plays a role as a strigolactone metabolic enzyme (Scaffidi et al., 2013).

Another scenario is that the high structural similarity imposes functional redundancy in the D14 family that affects SL or KL sensitivity and the resulting phenotypes. In this case, DLK2 could function as a SL/KL receptor that acts through the MAX2 core signaling pathway. Alternatively, parallel butenolide signaling pathways could interact, or DLK2 might mediate responses to an as yet unknown signal. Here we examine these hypotheses about DLK2 function and demonstrate that DLK2 is not involved in SL/KL perception and might act independently of the MAX2 pathway.

## Materials and Methods

### Plant Materials and Culture Conditions

Arabidopsis L*er* and Col-0 were used as wild types in this study. Seeds of mutants were obtained through the European Arabidopsis Stock Centre (NASC), except *dlk2-4* (L*er* background), which was isolated from the Cold Spring Harbor Ds collection (ET7593). Genotyping of *dlk2-4* was carried out with primer 1-primer 13 and primer 1–primer 12 (Supplementary Table S1). Mutant *dlk2-2* (SALK_068313C), *dlk2-3* (SALK_026193C; referred as D14L-2 by Toh et al., 2014), *d14-1* (WiscDsLoxHs137_07E), *kai2-2* (SGT6839) and *max2-2* lines were genotyped as described by Stirnberg et al. (2002) and Waters et al. (2012b). Mutant lines *htl-3*, *d14-1 htl-3, dlk2-3 htl-3* and *dlk2-3 d14-1 htl-3* (Toh et al., 2014) were kindly provided by David C. Nelson (University of California, Riverside, Riverside, CA, United States) and Peter McCourt (University of Toronto, Canada). Double mutant *d14-1 kai2-2*, and triple mutant *dlk2-3 d14-1 kai2-2* (six times backcrossed into Col-0) were kindly provided by Tom Bennett (University of Leeds, United Kingdom) and Mark Waters (University of Western Australia, Australia). Double mutant *dlk2-3 kai2-2* (gift from Tom Bennett) was outcrossed to Col-0 twice, and the resulting populations were screened for mutants. *pif*Q mutant (CS66049; Leivar et al., 2009) was obtained from NASC.

The plants were grown individually in Compo-Sana soil mixture in Conviron controlled environmental chambers with 16 h/8 h or 8 h/16 h photoperiod (80 μmol m^-2^ s^-1^, 21°C/18°C, 75% relative humidity).

### Strigolactone Compounds

GR24 racemic mixture (*rac*-GR24) was obtained from Chiralix (The Netherlands). Enantiopure (+)5DS and (-)5DS were purchased from OlChemim (Czech Republic). Compounds were diluted in either 100% acetone or DMSO.

### Hypocotyl Elongation and Cotyledon Expansion Assay

Seeds were surface sterilized in a solution containing 50% ethanol, 1.5% bleach and 0.05% (v/v) Tween20 for 10 min, then rinsed with 96% (v/v) ethanol and washed extensively with sterile water. Sterile seeds were cold stratified for 3 days (4°C). Stratified seeds were placed on solid 0.5 × MS medium (MS Basal Salt Mixture, pH 5.7; Sigma, United States) with 1% sucrose and the corresponding compound or DMSO (mock). To initiate germination, plates with the seeds were kept in red light (10 μmol m^-2^ s^-1^; LED) for 10 min. Plates were placed in SANYO (SANYO, Japan) controlled environmental chambers (21°C) and illuminated with continuous low intensity light (7 μmol m^-2^ s^-1^; fluorescent tubes). Seedlings were photographed at day 5 or 8 after germination. Captures were analyzed using ImageJ (National Institutes of Health, United States).

### General Molecular Biology

PCR amplifications were accomplished with Phusion DNA Polymerase (NEB, United States). cDNA for plasmid constructs was reverse transcribed with SuperScript III RT enzyme (Thermo Fisher Scientific, United States). GATEWAY compatible pGWB plasmids were kindly provided by Nakagawa et al. (2007). The binary vectors were generated by the standard GATEWAY procedure (Life Technologies, United States) using pDONR221 donor vector. Expression constructs were introduced into GV3101 Agrobacterium strain for floral dip transformation of Arabidopsis (Clough and Bent, 1998). T_3_-T_6_ transgenic plants were used in all experiments.

### *DLK2* Overexpression

To generate *DLK2* overexpressing and complementation lines in Col-0 (*dlk2-2*), complete cDNA was reverse-transcribed from Col-0 total RNA, then *DLK2* cDNA was amplified (Supplementary Tables S1, Primers 1 and 2a) and inserted into the *Nco*I-*BstE*II site of pCAMBIA1305.^1^ For DLK2 overexpressing lines in L*er* background [*DLK2* OE (L*er*)] with 6xHA tag (2x35Spro:cDLK2:6xHA), a secondary 35S promoter was inserted to the *Bam*HI-*Hind*III site of pCAMBIA1305. cDNA was amplified with primers containing a 6XHA-tag and STOP-codon (Supplementary Table S1, Primers 1 and 2b) and inserted into the *Nco*I-*BstE*II site of pCAMBIA1305-2x35S.

### GUS Histochemical Assay

To prepare DLK2pro:GUS (in Col-0 background), a 1023-bp genomic sequence including the promoter and 5’ UTR was amplified (Supplementary Table S1, Primers 3 and 4) and recombined into the pGWB533 binary vector. Plants harboring GUS constructs were grown either hydroponically in 0.5 × MS (pH 5.7) for 30 days, or on 0.5 × MS medium supplemented with equivalent amount of DMSO (mock) or *rac*-GR24. Samples were stained for 6 h according to the standard GUS protocol (Bomblies, 2000). To exclude positional effects, at least 10 parallel transformant lines were generated and only consistent patterns are discussed.

### Confocal Microscopy

For DLK2pro:DLK2:sGFP constructs, the same 1023-bp promoter region used for GUS constructs was used to drive the *DLK2* cDNA. Amplified fragments (Supplementary Table S1, Primers 3 and 6) were recombined into pGWB405 vector. For 35Spro:DLK2:sGFP, *DLK2* cDNA was amplified (Supplementary Table S1, Primers 6 and 7) and inserted into pGWB505. For microscopy experiments, five seedlings of four independent transgenic lines were removed from plates 14 days after sowing, placed on microscope slides covered with 0.5 × MS (solidified with 1% agar) and supplemented with either 0.01% DMSO (mock) or 10 μM *rac*-GR24. Samples were mounted in the same medium without agar under a cover glass and kept in a controlled environment between the measurements (10 μmol m^-2^ s^-1^, 21°C). A representative capture of GFP signal from mock and *rac*-GR24-treated seedling is presented. GFP signal was detected under a confocal microscope with the same exposure parameters at the excitation wavelength of 488 nm. Confocal imaging was carried out with a Leica TCS SP8 confocal laser scanning microscope (Leica, Germany).

### RNA Extraction and qRT-PCR Analysis

For RNA extraction, seedlings were grown as described in the Section “Hypocotyl Elongation and Cotyledon Expansion Assay.” The stratified seeds on plates intended for dark-grown seedlings were treated with red light for 10 min, kept in dark for 3 h, and then exposed to far red light for 10 min. All samples were harvested on day 4. Total RNA was isolated from at least 15 whole seedlings. RNA was isolated and DNAseI digested using RNEasy Plant Mini Kit (Qiagen, Germany). cDNA was reverse transcribed with Promega reverse transcriptase (Promega, United States). The qRT-PCR analyses were performed as described previously (Soós et al., 2012) with gene-specific *DLK2* primers (Waters et al., 2012b). The qRT-PCR results are presented as relative expression levels normalized against Arabidopsis *ACTIN2* (At3G18780; Hare et al., 2003; Mashiguchi et al., 2009; Zhou et al., 2014; Supplementary Table S1, Primers 14 and 15). All real-time PCR reactions were performed in quadruplicates, and means ± SD were calculated for three biological replicates for each examined treatment (*n* = 3, 15 seedlings in each).

### Protein Expression and Purification

Full-length coding sequences were amplified using Primers 8 and 9 for *DLK2* and Primers 10 and 11 for *D14* from L*er* cDNA (Supplementary Table S1), and were ligated into the *Nde*I and *Bam*HI or *Nde*I and *Eco*RI sites of pET-28c vector (Novagen, United States). Clones were sequenced and transformed into Rosetta DE3 pLysS cells (Novagen, United States). Protein expression was induced with 1 mM IPTG when the optical density at 600 nm reached 0.8, and incubated overnight (16 h) at 18°C/200 rpm. Harvested cultures were washed with NPI-10 buffer (50 mM NaH_2_PO_4_, 300 mM NaCl, 10 mM imidazole, pH 8.0) and stored at -80°C. Pellets were resuspended in NPI-10 buffer supplemented with 1 mg/mL lysozyme and 3 units/mL Pierce Universal Nuclease (Thermo Fischer Scientific, United States). Clarified lysates were batch purified using Protino Ni-NTA agarose beads (Macherey-Nagel, Germany). Bound proteins were eluted with NPI-250 buffer (50 mM NaH_2_PO_4_, 300 mM NaCl, 250 mM imidazole, pH 8.0), buffer-exchanged into 20 mM HEPES, pH 7.5, 150 mM NaCl, and 10% (v/v) glycerol and concentrated using Pierce Protein Concentrator (10 kDa; Thermo Fischer Scientific, United States). Protein concentration was estimated with Micro BCA Protein Assay Kit (Thermo Fischer Scientific, United States) and adjusted to 2 mg/mL. Protein purity was assessed by SDS-PAGE (Supplementary Figure S1).

### Thermal Shift Assay

The Differential Scanning Fluorimetry (DSF) was accomplished according to Niesen et al. (2007) and Waters et al. (2015b) with slight modifications. An Applied Biosystems 7500 Fast real-time PCR (Thermo Fischer Scientific, United States) was used to follow protein unfolding by monitoring the fluorescence of SYPRO Tangerine (Thermo Fischer Scientific, United States). Protein samples at 0.4 μg/μL (20 μM) in 100 mM HEPES buffer (pH 7.4) containing 150 mM NaCl, and 5% glycerol were screened in the presence and absence of different concentrations of (+)5DS, (-)5DS and DMSO. All reactions contained 5 × SYPRO Tangerine. Aliquots (10 μL) in four replicates were transferred to a 96-well PCR plate and scanned at a ramp rate of 1°C /min from 20 to 80°C. Curve fitting and melting temperatures were calculated using SimpleDSFViewer (Sun et al., 2015).

### *In Vitro* Hydrolysis Assay

Recombinant protein samples were thawed on ice. (+) or (-)5DS was added to 75 μL protein (80 μM) solution or buffer alone to a final concentration of 80 μM to achieve equimolar concentrations. Samples of mixture (20 μL) were taken immediately and after 2 h incubation at 22°C. Proteins were precipitated with 40 μL ice-cold acetone. From supernatants, 10 μL was injected into Agilent 1100 HPLC system (Agilent, United States) equipped with C18 reversed-phase column and combined with Waters SQ detector (Waters, United States). Samples were eluted under isocratic flow of 35% water and 65% acetonitrile. Peak areas of deoxystrigols at 235 nm were analyzed using MassLynx MS software (Waters, United States).

### Plant Protein Extraction and Western Blotting

Seedlings were grown for 2 weeks on 0.5 × MS agar plates then transferred to liquid 0.5xMS medium. Plants were incubated overnight at 22°C with gentle agitation (20 rpm). The seedlings were then transferred into liquid 0.5 × MS medium containing 10 μM *rac*-GR24 and incubated for 2, 6, 24 h in a growth chamber with gentle agitation. Seedlings were then blotted dry and snap-frozen in liquid nitrogen. Soluble proteins from approximately 100 mg of seedlings were extracted using 150 μL of lysis buffer (50 mM Tris, pH 7.5, 150 mM NaCl, 10% glycerol, 0.1% Tween 20, 1 mM PMSF, 1 mM DTT, and 1× Protease inhibitor cocktail). Lysates were clarified at 20,000 g for 10 min and supernatants were snap-frozen in aliquots. Protein concentration was estimated with Micro BCA Protein Assay Kit (Thermo Fischer Scientific, United States). Proteins were separated by 17.5% SDS-PAGE and blotted onto Hybond-LFP membrane (GE Healthcare, United States). Blots were blocked for 1 h at 22°C in TBS-T containing 5% casein. Primary antibodies [Mouse anti-GFP (clone: GF28R), dilution: 1:1000, Thermo Fisher Scientific, United States; mouse anti-actin (mABGEa), dilution: 1:1000, Thermo Fisher Scientific, United States] were diluted in TBS-T containing 1% casein. Membranes were incubated overnight at 4°C with primary antibodies then rinsed twice and washed twice for 10 min with TBS-T. Blots were incubated for 1 h on RT with secondary antibody (Goat anti-Mouse IgG (H + L) HRP conjugate, Thermo Fisher Scientific, United States) diluted 1:5000 in TBS-T. After rinsing two times and washing three times for 5 min with TBS-T, protein bands were visualized using 1-Step ultra TMB Blotting Solution (Thermo Fischer Scientific, United States) according to the manufacturers’ instructions.

### Statistical Analyses

ANOVA and *post hoc* comparisons of means with Tukey’s HSD test were performed with OriginPro software (OriginLab, United States).

## Results

### DLK2 Is Neither a Receptor, Nor a Hydrolase for SLs

**FIGURE 1:**
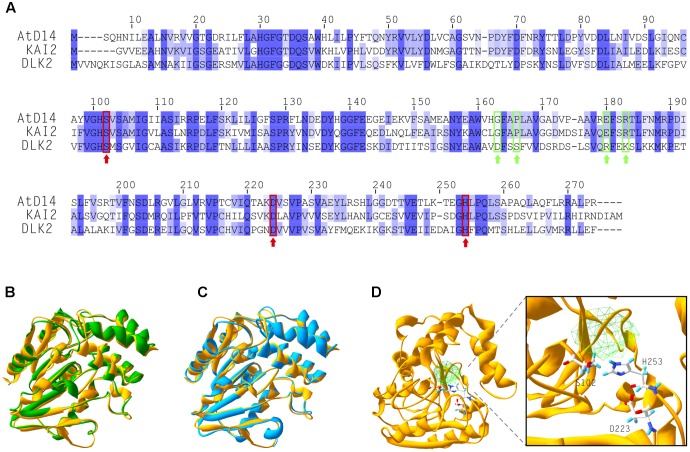
DLK2 shares high structural similarity with KAI2 and AtD14. **(A)** Alignment of amino acid sequences of AtD14, KAI2 and DLK2 showing that DLK2 shares 40 and 42% identity at the protein level with KAI2 and AtD14, respectively. Dark blue color shows identity in all three proteins; light blue coloring shows identity in two of the three proteins. The amino acids of the catalytic triad are marked with red rectangles and arrows. The residues required for the physical interaction of AtD14 with MAX2 are marked with green rectangles and arrows. **(B–D)** Crystallized tertiary structures of AtD14 (**B**, green; PDB code 4IH4), KAI2 (**C**, blue; PDB code 4IH1) and predicted structure of DLK2 (**D**, yellow; (I-TASSER server; http://zhanglab.ccmb.med.umich.edu/I-TASSER/). **(B,C)** The predicted structure of DLK2 (yellow) is overlaid on those of AtD14 and KAI2, respectively, using Swiss-PdbViewer (Guex and Peitsch, 1997). An expanded view of the catalytic triad residues of DLK2 (Ser-102, Asp-223 and His-253) and the predicted cavity are shown in **(D)**.

The protein structure – functional relationships of D14-family proteins have been studied in some detail (Hamiaux et al., 2012; Bythell-Douglas et al., 2013; Guo et al., 2013; Kagiyama et al., 2013; Nakamura et al., 2013; Zhao et al., 2013, 2015; de Saint Germain et al., 2016; Yao et al., 2016). DLK2 protein shares 40 and 42% amino acid sequence identity with KAI2 and AtD14, respectively (**Figure 1A**) and presumably evolved from D14 by gene duplication (Waters et al., 2012b). Several conserved sites can be aligned along the sequence, most importantly around the residues of the catalytic triad (**Figure 1A**). Interestingly, DLK2 lacks conserved amino acid residues in positions 163 (G), 166 (P), 180 (E) and 183 (R) (**Figure 1A**), which were shown to be essential for the interaction between AtD14 and D3 (Yao et al., 2016). The predicted structure of DLK2 was compared with crystal structures of AtD14 and KAI2 (**Figures 1B,C**) using the I-TASSER server. Based on the prediction, high structural similarity is present among the D14-family proteins. In particular, DLK2 contains a predicted ligand-binding pocket with the catalytic residues facing inward (**Figure 1D**), as is the case for its paralog proteins.

**FIGURE 2:**
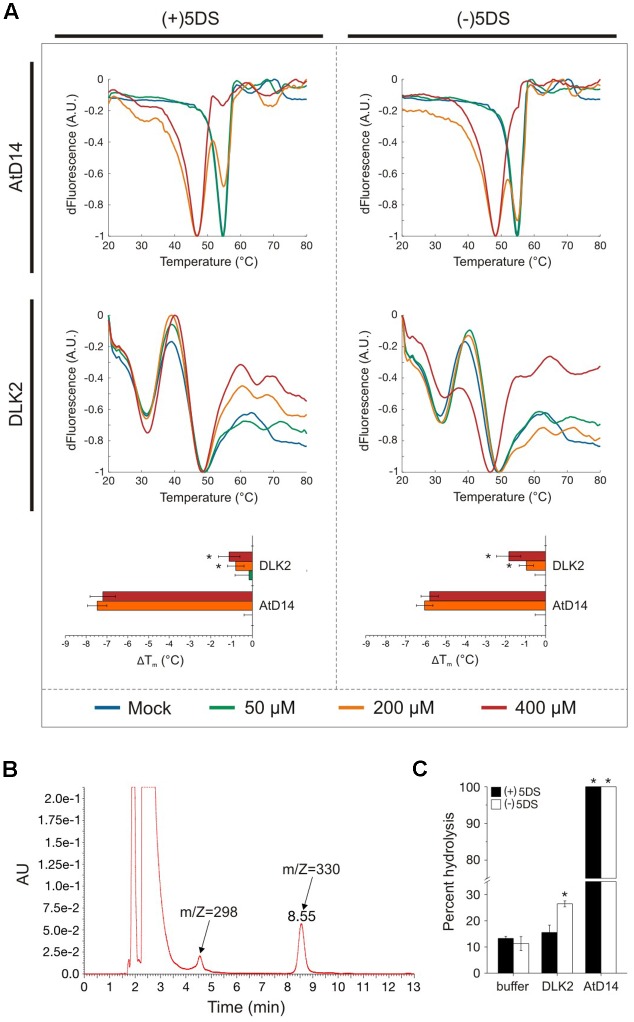
DLK2 stereospecifically binds and hydrolyses (–)5DS. **(A)** Differential Scanning Fluorimetry (DSF) assay curves of AtD14 and DLK2 proteins (purified from *E. coli* Rosetta cells) in the presence of (+) and (–)5DS. Protein-ligand mixtures with SYPRO fluorescent dye were gradually heated in a real-time PCR instrument and the change in fluorescence emission was monitored and plotted against temperature. Curve fitting was accomplished with SimpleDSFviewer (Sun et al., 2015). ΔT_m_ is calculated as a difference from mock (DMSO) T_m_; calculated T_m_ values of three protein samples and four technical replicates from different protein batches are shown; asterisks represent a significant difference from mock (buffer) under the same conditions (mean ± SD; ANOVA, *P* < 0.01). **(B)** (+)5DS and (–)5DS (m/Z = 330) show emission peaks at 235 nm. Peak of the putative degradation product is also shown (m/Z = 298). Amounts of deoxystrigols and degradation products were estimated as the areas of peaks. **(C)** Hydrolysis of (+) and (–)5DS in the presence of AtD14 and DLK2. Hydrolysis of the compounds was assessed by HPLC after 2 h of incubation at 20°C. Data represent the percentages of decrease in the peak areas of substrates. The spontaneous hydrolysis was around 10%. Measurements were repeated at least three times using different protein batches. Asterisks represent significant differences from mock (buffer) under the same conditions (mean ± SD; ANOVA, *P* < 0.01).

The presence of the conserved catalytic triad prompted us to test whether DLK2 also binds and/or hydrolyses SLs in *in vitro* assays using recombinant proteins expressed in *Escherichia coli*. DSF has been established as a reliable method to infer alterations of protein thermal stability in the presence of a small-molecule interaction partner (Niesen et al., 2007). In particular, DSF assays have been used to characterize the melting temperature (T_m_) shifts of DAD2, AtD14 and KAI2 in the presence of SLs (Hamiaux et al., 2012; Waters et al., 2015b). DSF data obtained from the positive control AtD14 was consistent with the previous findings (Waters et al., 2015b), exhibiting a significant ligand concentration-dependent lowering of T_m_ in the presence of SLs regardless of the stereochemistry of the ligands (**Figure 2A**). Under all conditions tested, DLK2 exhibited a characteristic two-phase melting curve, suggesting that DLK2 either has two distinct phase transitions or can be present in monomer and dimer forms (**Figure 2A**; Fang et al., 2010; Silva et al., 2012). Addition of (+)5DS did not cause a T_m_ shift for DLK2; however, in the presence of the highest concentration of (-)5DS, T_m_ shifted moderately (2°C) lower (ANOVA, *p* < 0.01) implying that it can bind to DLK2 and destabilize it (**Figure 2A**).

Having demonstrated a stereospecific interaction between (-)5DS and DLK2, we tested the proposed hydrolytic function of DLK2 *in vitro*. The consumption of (+)5DS and (-)5DS and the production of a possible metabolite were monitored by HPLC (**Figure 2B**), as described by Waters et al. (2015b) with minor modifications. While SLs hydrolyzed spontaneously at a 10%/h rate, AtD14 hydrolyzed 100% of both substrates in 2 h (**Figure 2C**). Consistent with the thermal stability assay, DLK2 exhibited moderate hydrolytic activity only against the non-natural enantiomer (-)5DS (**Figure 2C**). Stereospecific binding and hydrolysis suggests that recombinant DLK2 is not a receptor of tested SLs. In terms of hydrolytic activity and affinity toward the stereoisomers of deoxystrigol, DLK2 more closely resembles KAI2, which specifically binds and hydrolyzes only (-)5DS (Waters et al., 2015b), suggesting that both proteins might have a non-SL butenolide ligand. Furthermore, as DLK2 does not hydrolyze natural SL (+)5DS, and only slowly hydrolyzes non-natural (-)5DS, we can conclude that DLK2 is not a SL metabolism enzyme as had been suggested by the positive feedback regulation of DLK2 expression (Scaffidi et al., 2013).

### *DLK2* Overexpression Results in Elongated Hypocotyls

Binding and hydrolysis of the non-natural (-)5DS by DLK2 shows similarity to the same properties of KAI2 (Waters et al., 2015b), raising the question whether the two proteins might interfere within the plant, possibly having the same natural ligand and redundantly regulating developmental responses. Thus, we tested whether absence or overexpression of *DLK2* results in any MAX2-related phenotypic alterations, and if so, whether crosstalk between the three D14 family related pathways is manifest in the phenotypes. *dlk2* mutants in Col-0 background were reported to be normal with respect to seed dormancy, germination and shoot branching phenotypes (Waters et al., 2012b). We assessed these traits and other SL-related phenotypes such as senescence and branching in *dlk2* mutants (in L*er* and Col-0 background) as well as in *DLK2*-overexpressing lines (OE). No obvious phenotypic differences in branching were observed in adult OE lines and mutant plants growing in long days and in rosettes grown in short days (Supplementary Figure S2), nor in progress of senescence (Supplementary Figure S3) or seed germination characteristics (Supplementary Figure S4).

**FIGURE 3:**
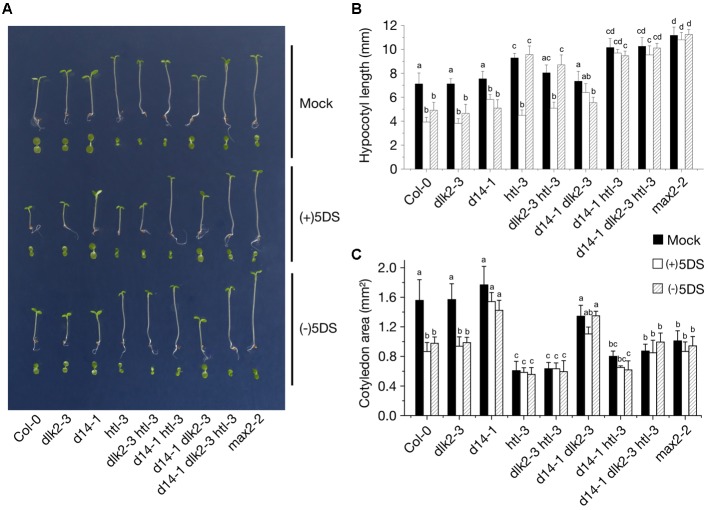
Seedling growth responses to low light conditions of the *dlk2-3* mutant and its combinations with *htl-3* and *d14-1* mutants. **(A)** Seedling phenotypes of 5-day-old *dlk2-3*, *d14-1* and *htl-3* mutants and their combinations either untreated or exposed to 10 μM of (+)5DS and (–)5DS. Seeds were sown on 0.5XMS plates with 1% sucrose and supplemented with 10 μM of each compound as indicated. To initiate germination, seeds were dark stratified and were kept in red light (10 μmol m^-2^ s^-1^; LED) for 10 min. Plates were incubated for 5 d under low light conditions (7 μmol m^-2^ s^-1^; 21°C). **(B,C)** Hypocotyl elongation **(B)** and cotyledon expansion **(C)** responses of *dlk2-3*, *d14-1* and *htl-3* mutants and their combinations to 10 μM of (+)5DS and (–)5DS applications as compared to wild type Col-0 seedlings grown in low light for 5 days. Data are means of 3 independent experiments, >30 seedlings in each. Bars with the same letter are not significantly different from each other (mean ± SD; ANOVA, *P* < 0.01, Tukey’s HSD test).

MAX2 acts as a promoter of seedling photomorphogenesis (Shen et al., 2007). To investigate whether DLK2 is involved in these MAX2-related signaling events, we tested *dlk2* seedling responses to suboptimal light conditions, when the effect of *max2* mutation is more prominent (Stirnberg et al., 2002; Shen et al., 2007). Previous work found that Arabidopsis *kai2* mutant seedlings showed distinct photomorphogenic phenotypes compared to wild type (L*er*), while *dlk2* mutants did not (Waters et al., 2012b). Consistent with this finding, *dlk2* mutants in Col-0 and L*er* backgrounds exhibited normal photomorphogenic responses under low light conditions (**Figure 3** and Supplementary Figure S5).

To test whether functional redundancy exists among D14-family proteins affecting seedling phenotypes and SL sensitivity, we examined double and triple mutants of *dlk2-3*, *d14-1*, *htl-3* (a *kai2* allele in Col-0) (**Figure 3**) and *kai2-2* (L*er* background; Supplementary Figure S5), grown under continuous low intensity white light in which mutants display distinct hypocotyl and cotyledon growth responses. Untreated *dlk2-3*, *dlk2-4* and *d14-1* single mutants seedlings displayed no significant differences from their wild types in hypocotyl elongation and cotyledon expansion, while *htl-3* (*kai2-2*) single mutants exhibited significantly greater hypocotyl elongation and decreased cotyledon expansion under low intensity light (**Figures 3A–C** and Supplementary Figure S5), consistent with several previous reports (Waters et al., 2012b; Scaffidi et al., 2013; Toh et al., 2014). Seedlings of the double mutant *dlk2-3 htl-3, d14-1 htl-3* and the triple mutant *d14-1 dlk2-3 htl-3* displayed increased hypocotyl length similar to those of the single *htl-3* mutant, confirming that *KAI2* contributes to inhibition of hypocotyl elongation in response to light (**Figures 3B,C**). However, untreated *dlk2-3 htl-3* (and *dlk2-3 kai2-2*) seedlings exhibited slightly shorter hypocotyls than *htl-3 (kai2-2)* seedlings, implying that DLK2 might be involved in the promotion of hypocotyl elongation by low light.

Differences in inhibition of hypocotyl elongation by SLs were also evident among the mutant seedlings. Both (+)5DS and (-)5DS inhibited elongation of *dlk2-3* mutant hypocotyls, while the *htl-3* mutation alone or in combination with *dlk2-3* exhibited growth inhibition only in the presence of (+)5DS (**Figure 3B**). When combined with *d14-1*, the *htl-3* mutation resulted in loss of sensitivity to both (+)5DS and (-)5DS, and presence of the *dlk2* mutation did not affect substantially these effects of *d14-1* and *htl-3* (and *kai2-2*) (**Figure 3** and Supplementary Figure S5).

In untreated seedlings, the *dlk2-3* and *d14-1* mutations alone or together had no effect on cotyledon expansion while the *htl-3* (and *kai2-2*) mutation in any combination reduced expansion by 40–70% (**Figures 3A,C** and Supplementary Figure S5). Similar to the action of racemic GR24 (*rac*-GR24) (Waters et al., 2012b), (+)5DS and (-)5DS inhibited cotyledon expansion of Col-0 and L*er* wild types while lines containing *d14-1* (and *d14-1* plus *dlk2-3*) were insensitive to these SLs (**Figures 3A,C** and Supplementary Figure S5). All multiple mutant lines containing *htl-3* exhibited reduced cotyledon expansion similar to the *htl-3* single mutant, implying that KAI2 is the primary promoter of cotyledon expansion.

**FIGURE 4:**
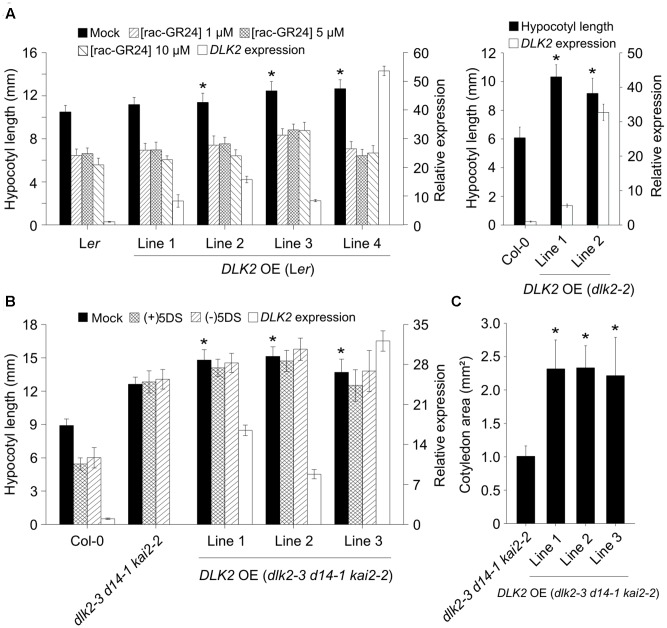
*DLK2* overexpression (OE) lines exhibit elongated hypocotyl response to low light conditions. **(A)** Hypocotyl elongation responses of low light grown *DLK2* OE (L*er*) and *DLK2* OE (*dlk2-2*) lines (10 days old). Seeds were germinated and grown on 0.5 × MS plates with 1% sucrose and supplemented with 1, 5, and 10 μM of *rac*-GR24 applications [*DLK2* OE (L*er*)] as compared to wild type seedlings. Data are means of 5 independent experiments, >30 seedlings in each. Asterisks represent a significant difference from L*er* or Col-0 under the same treatment (mean ± SD; ANOVA, *P* < 0.025). Real-time PCR was corroborated in all lines with three biological replicates (*n* = 3, 15 10-day-old seedlings in each; Col-0 was set as calibrator); reactions were performed in quadruplicates. **(B)** Hypocotyl elongation responses of *DLK2* OE (*dlk2-3 d14-1 kai2-2*) lines. Seeds were germinated and grown on 0.5 × MS plates with 1% sucrose and supplemented with 10 μM of (+)5DS and (–)5DS. Data are means of 3 independent experiments, >30 seedlings in each. Asterisks represent a significant difference from *dlk2-3 d14-1 kai2-2* under the same treatment (mean ± SD; ANOVA, *P* < 0.025). Real-time PCR was corroborated in all lines with three biological replicates (*n* = 3, 15 10-day-old seedlings in each; Col-0 was set as calibrator); reactions were performed in quadruplicates. **(C)** Cotyledon expansion responses of *DLK2* OE (*dlk2-3 d14-1 kai2-2*) lines. Seeds were germinated and grown on 0.5 × MS plates with 1% sucrose. Data are means of 3 independent experiments, >30 seedlings in each. Asterisks represent significant differences from *dlk2-3 d14-1 kai2-2* under the same treatment (mean ± SD; ANOVA, *P* < 0.025).

Overexpression of *DLK2* driven by a 2 × 35S promoter in L*er* plants [*DLK2* OE (L*er*)] or by a 35S promoter in *dlk2-2* [*DLK2* OE (*dlk2-2*)] exhibited longer hypocotyls compared to wild type controls grown for 8 days under low light conditions (**Figure 4A**). This finding suggests that DLK2 might promote hypocotyl elongation in low light. To test whether DLK2 dose might counteract SL inhibition on hypocotyl elongation, *DLK2* OE (L*er*) plants were subjected to treatment with increased concentrations of SLs. However, *rac*-GR24-induced suppression of hypocotyl elongation was not affected by *DLK2* overexpression (**Figure 4A**).

It was reported that *DLK2* is downregulated in *d14 kai2* background (Waters et al., 2012b); thus, this double mutant might be regarded as a functional *dlk2* mutant as well. To examine any possible phenotypes related to DLK2 and to exclude the effects of its paralogs, we generated *DLK2* OE lines in the triple mutant background [*DLK2* OE (*dlk2-3 d14-1 kai2-2*)] with the construct 35Spro:DLK2:sGFP. We found that DLK2 overexpression resulted in a slightly more elongated hypocotyl in *dlk2-3 d14-1 kai2-2* mutants (**Figure 4B**). Furthermore, these plants exhibited more pronounced cotyledon expansion than triple mutants (**Figure 4C**). As DLK2 binds and weakly hydrolyzes (-)5DS *in vitro*, we tested whether the compound would inhibit growth of *DLK2* OE (*dlk2-3 d14-1 kai2-2)* hypocotyls. *DLK2* OE (*dlk2-3 d14-1 kai2-2*) lines were unresponsive to both (+)5DS and (-)5DS, indicating that DLK2 does not transduce (-)5DS signal (**Figure 4B**).

### *DLK2* Upregulation in the Dark Is Dependent upon KAI2 and PHYTOCHROME INTERACTING FACTORS (PIFs)

**FIGURE 5:**
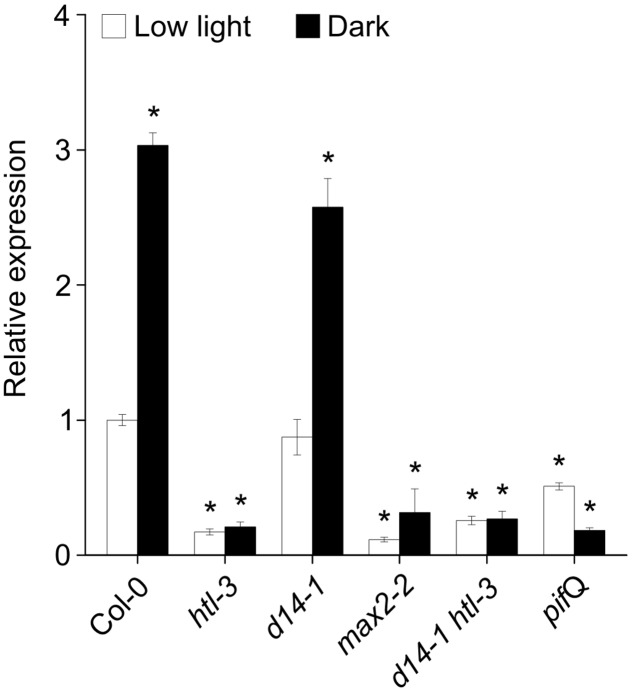
*DLK2* transcripts are upregulated in the dark through KAI2 and PIFs. Dark adaptation upregulates *DLK2* in 5-day-old dark-grown seedlings. Seeds were germinated and grown on 0.5 × MS plates with 1% sucrose. Real-time PCR was conducted on three biological replicates (*n* = 3, 15 seedlings in each). Asterisks represent significant differences from Col-0 (low light; mean ± SD; ANOVA, *P* < 0.025). Reactions were performed in quadruplicates.

*DLK2* expression is highly dependent on the D14 and KAI2 signaling pathways, as extremely low expression was detected in either *d14 kai2* or *max2* mutants (Waters et al., 2012b). *DLK2* transcription was induced by (+)5DS through AtD14 and by (-)5DS through KAI2 (Scaffidi et al., 2013), but unlike *KAI2* and *AtD14*, *DLK2* expression was not regulated by light (Waters et al., 2012b). However, we observed that *DLK2* expression was significantly upregulated in Col-0 and *d14-1* seedlings grown for 5 days in the dark (**Figure 5**). This upregulation was absent when the *htl-3* mutant was present and in *max2-2* single mutant seedlings, suggesting that light may modulate *DLK2* expression through KAI2 and that the response of *DLK2* to dark/light is dependent on MAX2-related signaling (**Figure 5**). We also tested whether PIF proteins, transcription factors promoting skotomorphogenesis (Leivar et al., 2009) are required for *DLK2* expression. We found that *DLK2* transcript abundance was lower in quadruple *pif* (*pif*Q) mutants growing in light and *DLK2* expression was not induced by dark in *pif*Q mutants, demonstrating that *DLK2* expression is directly affected by light response pathways.

### *DLK2* Expression Pattern Is Affected by SLs and Dark

**FIGURE 6:**
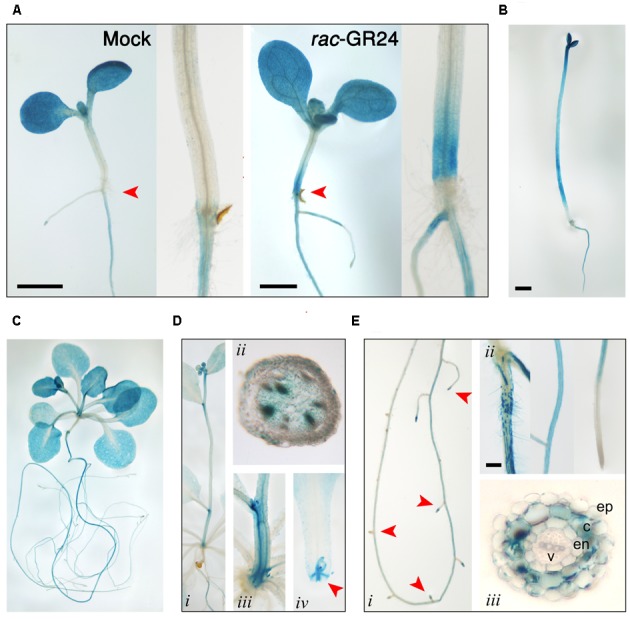
Spatio-temporal regulation of *DLK2* promoter activity in DLK2pro:GUS transgenic seedlings and adult plants (bars = 1 mm). **(A)** GUS histochemical activity in mock and *rac*-GR24 (10 μM) treated 10-day-old seedlings grown under low light conditions (7 μmol m^-2^ s^-1^). Insets show close-ups of the basal section of the hypocotyls. **(B)** GUS histochemical activity in 6 days old dark grown seedlings. **(C)** GUS histochemical activity in 4 weeks old whole plants. **(D)** GUS histochemical activity in the aerial parts of 4 weeks old plants. (i) Stem (ii) Hand section of a stem segment adjacent to the first cauline leaf. (iii) Close-up of the basal part of stem. (iv) Close-up of the basal part of the petiole with the stipules and bud. **(E)** GUS histochemical activity in the roots of 4 weeks old plants. (i) Whole root (ii) Root segment close to the hypocotyl root junction; root in the differentiation zone; root cap. (iii) Hand section of the root in the differentiation zone.

To elucidate the spatio-temporal regulation of *DLK2* expression induced by dark and SLs, we generated a transcriptional fusion of a 1023 bp DLK2 promoter fragment with the GUS gene-coding region. We assayed for GUS expression in at least seven representative T_4_ homozygous Arabidopsis Col-0 lines. In young control seedlings grown on 0.5 × MS plates, GUS stain was detected first in the cotyledons which progressively intensified with the onset of the cotyledon expansion and subsequently was detected also in the roots (**Figure 6A**). In seedlings grown on plates supplemented with 10 μM *rac*-GR24, a specific and strong GUS signal appeared at the basal end of the hypocotyl (**Figure 6A**). In accordance with the real-time PCR data, dark-grown seedlings displayed intensive GUS accumulation (**Figure 6B**), especially in the hypocotyl. In the aerial parts of adult plants, GUS signal was strong in primary and mature leaves and petals (**Figures 6C,D**). No GUS activity was detected in mature hypocotyl, petiole vasculature and non-elongating, mature stems (**Figures 6C,D**), while the axillary buds and the vascular bundles of elongating stem segments adjacent to the cauline leaves displayed intensive GUS staining (**Figures 6C,D**). Interestingly, *DLK2* promoter activity was strong in buds and the vascular cells connecting the stipules with the vasculature of the petiole (**Figure 6D**). In the root system of adult plants, GUS activity was strong in the differentiation zone and the GUS signal gradually faded away toward the primary root tip (**Figures 6C,E**). *DLK2* promoter activity was the strongest in root hairs and in the cortex (**Figure 6E**) of adult plants. Notably, lateral root primordia displayed no GUS signal, while *DLK2* promoter activity was detected in young lateral root tips (**Figure 6E**). These findings indicate that *DLK2* expression pattern is tissue specific and regulated by SLs or dark directly.

### DLK2 Is Not a Subject of *rac*-GR24-Mediated Protein Degradation

**FIGURE 7:**
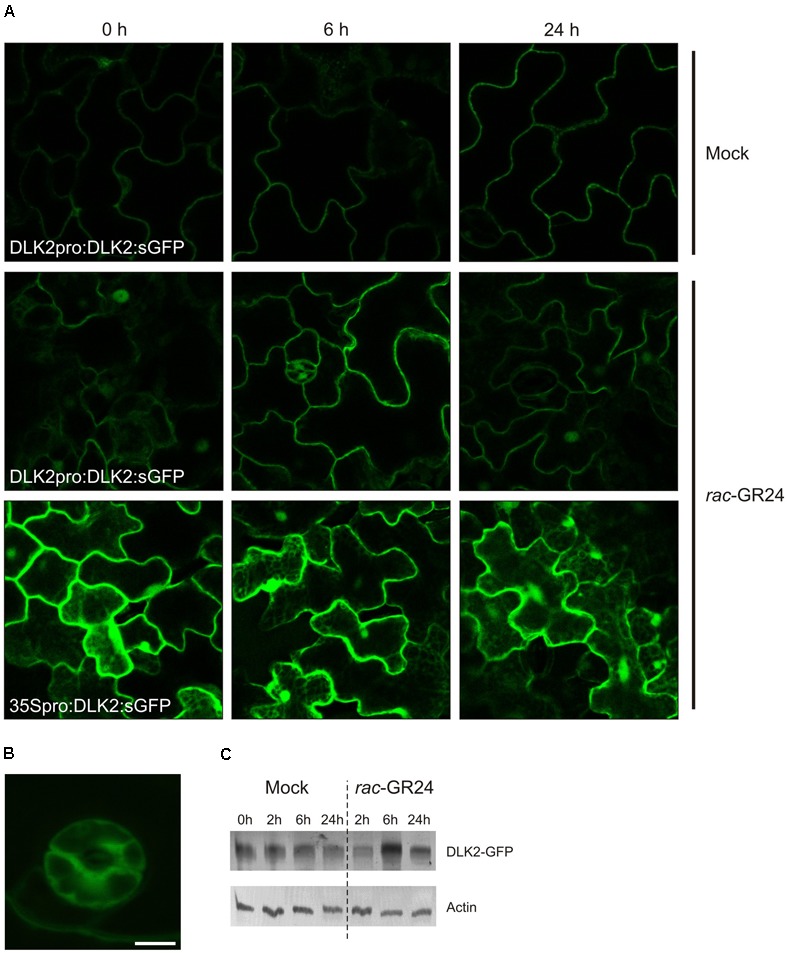
DLK2 does not show *rac*-GR24-specific degradation. **(A)** Representative captures show that DLK2:sGFP accumulates upon *rac*-GR24 treatment in the primary leaves of 14 days old DLK2pro:DLK2:sGFP plants. GFP is detected in the cytoplasm and nucleus. **(B)** DLK2-sGFP expression in guard cells of the primary leaves of 14 days old DLK2pro:DLK2:sGFP plants (bar = 5 μm). **(C)** Two weeks old seedlings of 35Spro:DLK2:sGFP were treated with 10 μM *rac*-GR24 for 2, 6, 24 h. Total protein was extracted, run on SDS-PAGE and blotted. DLK2-sGFP fusion protein was visualized using GFP specific antibodies. DLK2-sGFP protein levels show slight increase during *rac*-GR24 treatment compared to untreated samples.

To assess whether DLK2 is degraded upon SL treatment and to further characterize *DLK2* expression in tissues and at the subcellular level, we generated translational fusions of *DLK2* cDNA to sGFP, driven by the 1023-bp *DLK2* promoter in DLK2pro:DLK2:sGFP or DLK2 cDNA driven by the constitutive CaMV35S promoter in a 35Spro:DLK2:sGFP construct. Consistent with the PSORT prediction (Horton et al., 2007), DLK2 localizes in the cytoplasm and nucleus (**Figure 7A**). In DLK2pro:DLK2:sGFP plants, a strong GFP signal has been observed in guard cells (**Figure 7B**). When plants harboring a *DLK2* promoter-driven sGFP construct (DLK2pro:DLK2:sGFP) were subjected to *rac*-GR24 treatment, a transient increase in GFP signal intensities was detected, while the constitutive 35S promoter resulted in higher GFP expression in the epidermal cells that did not change in response to *rac*-GR24 (**Figure 7A**). Consistent with this, immunoblot assays using constitutively expressing lines (35Spro:DLK2:sGFP) showed that DLK2 was not targeted for degradation after *rac*-GR24 treatments (**Figure 7C**). Instead, DLK2 protein slightly accumulated after 6 h in *rac*-GR24-treated 21-day-old whole 35Spro:DLK2:sGFP plants (**Figure 7C**). In DLK2pro:DLK2:sGFP plants, DLK2 accumulated upon *rac*-GR24 treatments (**Figure 7A**) confirming that SLs induce upregulation of *DLK2* as shown earlier (Waters et al., 2012b; Scaffidi et al., 2014). These findings suggest that unlike AtD14 (Chevalier et al., 2014) or KAI2 (Waters et al., 2015a), DLK2 protein degradation is not promoted by *rac*-GR24 SLs (**Figures 7A,C**), instead, DLK2 remains stable upon *rac*-GR24 treatment.

## Discussion

There is compelling evidence that at least two butenolide signaling pathways exist in vascular plants. The ancient KAI2 pathway has an as yet unknown butenolide ligand (KL; Conn and Nelson, 2016; Sun et al., 2016; Waters, 2017), which is neither SL nor karrikin. During the course of evolution, KAI2 underwent a gene duplication event which resulted in the D14 clade (Delaux et al., 2012; Bennett and Leyser, 2014). The D14 pathway perceives the canonical SL ligand and diverged from the KAI2 clade both evolutionarily and physiologically (Waters et al., 2012b). The question then emerges, how does DLK2 relate to these MAX2-dependent signaling pathways? We showed that recombinant DLK2 does not hydrolyze (+)5DS and is not destabilized in the presence of (+)5DS (**Figure 1**), indicating that DLK2 is not an SL receptor nor an SL hydrolase that functions in a negative feedback system to remove excess SL. This is further supported by the sensitivity of *dlk2* mutants to (+)5DS (**Figure 3**) and *rac*-GR24 (Waters et al., 2012b) and *DLK2* OE lines do not show a SL-deficient phenotype (Supplementary Figures S2, S3). On the other hand, compared to AtD14, DLK2 shows weaker stereospecific binding and hydrolysis toward (-)5DS (**Figure 1**), a non-natural SL which, along with karrikins, oddly substitutes for the unknown endogenous KAI2 ligand (Conn and Nelson, 2016). It is intriguing to consider that DLK2 might be a receptor or hydrolase for the enigmatic KL(s). The structure of KL is unknown; therefore, it is hard to draw a parallel between DLK2 and KAI2 ligand-binding mechanisms, and SL binding does not necessarily result in physiological effects (Waters et al., 2015b; de Saint Germain et al., 2016). The light hyposensitivity of *DLK2* overexpressing lines (**Figure 4**) might be the consequence of KL metabolism by excess DLK2 and the elongated hypocotyl phenotype of *DLK2* OE lines resembles the *htl-3* (*kai2-2*) hypocotyl phenotype, however, other *htl-3*-related traits, such as suppressed cotyledon expansion or broad leaves (Waters et al., 2012b, 2015b, Soundappan et al., 2015) were not observed in these lines. Furthermore, *dlk2* mutants are sensitive to (-)5DS (**Figure 3**) and to karrikin treatment (Waters et al., 2012b), suggesting that DLK2 is not involved in KL signaling, although (-)5DS and karrikin do not necessarily mimic KL action. We propose that DLK2 neither perceives nor hydrolyzes the natural ligand of D14 and KAI2. A remaining question is whether DLK2 should be regarded as a component of a separate signaling pathway, or is its function merely to regulate other MAX2-dependent pathways through the sequestration of the signaling molecules.

The known pathways related to the D14 family diverge at the level of SMXL-family proteins. Intuitively, the weakly characterized members of the SMXL/D53 family, SMXL3, -4 and -5 might be co-opted by DLK2. SMXL4, originally referred to as AtHSPR (*Arabidopsis thaliana* HEAT SHOCK PROTEIN-RELATED), plays a role in abiotic stress responses (Yang et al., 2015) and displays a vascular bundle-specific expression (Zhang L. et al., 2014; Zhang Y. et al., 2014), as does *DLK2* in elongating stem segments. It was shown recently that *smxl4 smxl5* double mutants are defective in carbohydrate accumulation and phloem transport (Wu et al., 2017) and SMXL3, -4 and -5 are essential for phloem formation (Wallner et al., 2017). In SMXL3, -4 and -5, the RGKT motif needed for MAX2-mediated protein degradation of D53/SMXL7 is absent (Soundappan et al., 2015), and SMXL5 is not degraded upon *rac*-GR24 application (Wallner et al., 2017), suggesting that these proteins may not be degraded through MAX2. Intriguingly, DLK2 lacks the residues required for the physical interaction with MAX2. A recent publication also suggested that DLK2 homologues presumably do not interact with MAX2 (Bythell-Douglas et al., 2017). The glycine residue in position 158 is required to form a π-turn structure, which is a prerequisite of proper conformational changes of the D14 lid during SL activation (Yao et al., 2016). Other substitutions that reportedly do disrupt D14–MAX2 interactions (P161D, E174A, R177A; Yao et al., 2016), and are conserved in KAI2, are not present in DLK2 (**Figure 1**). Furthermore, DLK2 is not degraded upon *rac*-GR24 application (**Figure 7**) suggesting that DLK2 does not interact with MAX2; however, its expression regulation is mostly accomplished through MAX2 (**Figure 5**). It was previously shown that upon binding their proposed ligand, AtD14 and KAI2 underwent substrate-induced protein degradation. AtD14 is degraded in a MAX2-dependent manner through the 26S proteasome system (Chevalier et al., 2014), and KAI2 is degraded independently of MAX2 and 26S proteasomes (Waters et al., 2015a). The immunoblot analysis showed a slight increase in the amount of DLK2:sGFP protein even in 35Spro:DLK2:sGFP plants, suggesting a posttranscriptional effect (**Figure 7C**).

It cannot be ruled out that other butenolides or the proposed KL might promote DLK2 degradation. A potential future direction of DLK2 research could be the elucidation of the relationship between DLK2 and SMXL3, SMXL4, and SMXL5.

We demonstrated that KAI2 is a principal promoter of cotyledon expansion in the D14 family, although interactions can be observed. Overexpression of *DLK2* in wt, *dlk2-2* and *dlk2-3 d14-1 kai2-2* backgrounds results in more elongated hypocotyls and (in the case of triple mutant) expanded cotyledons under low light conditions (**Figure 4**), suggesting that DLK2 is indeed capable of regulating these physiological responses *per se*. However, *dlk2* mutants do not display the opposite phenotypes, and the phenotype of the OE lines does not correlate with the transcript level (**Figure 4A**), so neomorphic or hypermorphic effects of DLK2 overexpression cannot be ruled out. We propose that DLK2 can promote hypocotyl elongation under suboptimal light conditions, although this effect is modulated by other members of the D14 family. This finding is in conflict with the interpretation of an earlier report (Kameoka and Kyozuka, 2015), where the authors suggested that the shorter mesocotyls of *KAI2*-RNAi *d14* seedlings compared to those of the *d3* mutant in rice is due to suppression by DLK2. However, differences between species might also contribute to this effect, and, as the authors noted, this finding should be interpreted with caution as there was residual *KAI2* expression in the RNAi lines.

We demonstrated that apart from the well documented SL and karrikin responsiveness, *DLK2* expression is also down-regulated by light. Dark adaptation promotes *DLK2* expression especially in the hypocotyl, and *DLK2* upregulation in dark-kept seedlings is accomplished through MAX2 and KAI2 (**Figures 5**, **6**). *DLK2* expression is suppressed in the *pif*Q mutant either in light or dark, indicating that light signaling regulates *DLK2* transcription via PIFs. It is also noteworthy that the spatial *DLK2* expression pattern is regulated by *rac*-GR24 (**Figure 6**), suggesting a dynamic adaptation of *DLK2* transcription to hormonal and environmental changes. *DLK2* activity is strong in root hair and cortex, implying that DLK2 might be involved in the physiological processes linked to these tissues, such as water and nutrient uptake (Tanaka et al., 2014) and edaphic stress responses (Schneider et al., 2017). *DLK2* expression was strong in axillary buds and the adjacent vascular bundles might also suggest that DLK2 plays a role in the regulation of nutrient distribution.

In summary, the results herein show that although it is structurally similar to its paralog D14 family proteins, DLK2 only weakly binds or hydrolyzes natural and unnatural SL ligands. DLK2 is widely expressed in seedlings and has a role in the promotion of hypocotyl elongation. These data together with the knowledge accumulated so far on DWARF14 family suggest that DLK2 represents a divergent member of the family. The fine details of DLK2 regulation, signaling and its role in adult plant life are the subject of future investigations.

## Author Contributions

AV, NI, and VS designed and performed most of the experiments, conceived the project and wrote the paper, AF performed experiments with confocal microscopy, HH and KB contributed to conception and design of the experiments and to editing of the text, EB conceived the project and supervised writing.

## Conflict of Interest Statement

The authors declare that the research was conducted in the absence of any commercial or financial relationships that could be construed as a potential conflict of interest.

## Footnotes

1 http://www.cambia.org/daisy/cambia/585.html
