# Supplementary material for: Comprehensive Analysis of DWARF14-LIKE2 (DLK2) Reveals Its Functional Divergence from Strigolactone-Related Paralogs
Source: Front Plant Sci. 2017 Sep 22;8:1641. doi: 10.3389/fpls.2017.01641 (PMC5609103; doi:10.3389/fpls.2017.01641)
Supplement: Supplementary file 1 [file Table_1.PDF]

Supplemental Table 1.

Primer ID	Primer Name	Sequence
Primer 1	AtDcDNANcolF	5'-GCCCATGGTGGTTAATCAGAAGATATC-3'
Primer 2a	AtDcDNABstEII6xHAR	5'-CGGGTCACCTTAAGCGTAATCTGGAACATCGTATGGGTAAGCGTAATC
		TGGAACATCGTATGGGTACCTAGGAAACTCAAGGAGGCGCCTCATGA-3'
Primer 2b	AtDcDNABstEII	5'- CGGGTCACCTTAAAACTCAAGGAGGCGCCTCATGA -3'
Primer 3	AtDLK21kpF	5'-GGGGACAAGTTTGTACAAAAAAGCAGGCTTCATACACTTTTAAATACCAGTTTC-3'
Primer 4	AtDLK22kpR	5'-GGGGACCACTTTGTACAAGAAAGCTGGGTCGCTTAAGTACAAGAGTTTTGTTA-3'
Primer 5	AtDLK22kipR	5'-GGGGACCACTTTGTACAAGAAAGCTGGGTCATTTGCATTTCAATCTAGTAAC-3'
Primer 6	AtD2cDNAattbR	5'-GGGGACCACTTTGTACAAGAAAGCTGGGTCAAACTCAAGGAGGCGCCTCAT-3'
Primer 7	AtD2cDNAattbF	5'-GGGGACAAGTTTGTACAAAAAAGCAGGCTTCATGGTGGTTAATCAGAAGATA-3'
Primer 8	AtDLK2Ndelfw	5'-GGAATTCCATATGGTGGTTAATCAGAAG-3'
Primer 9	AtDLK2BamHISTOPrev	5'-TTAGGATCCTTAAAACTCAAGGAGGCGCCT-3'
Primer 10	AtD14Ndelfw	5'-GGAATTCCATATGAGTCAACACAACATCTT-3'
Primer 11	AtD14EcoRISTOPrev	5'-CCGGAATTCTCACCGAGGAAGAGCTCGCC-3'
Primer 12	AtDwR	5'-TGGAATGTTTTATTCAACAACAAC-3'
Primer 13	Ds3	5'-ACCCGACCGGATCGTATCGGT-3'
Primer 14	ACT2F	5'-CTTGCACCAAGCAGCATGAA-3'
Primer 15	ACT2R	5'-CCGATCCAGACACTGTACTTCCTT-3'

Supplemental Table 1. Primer sequences used in this study

Supplemental Figure S1.

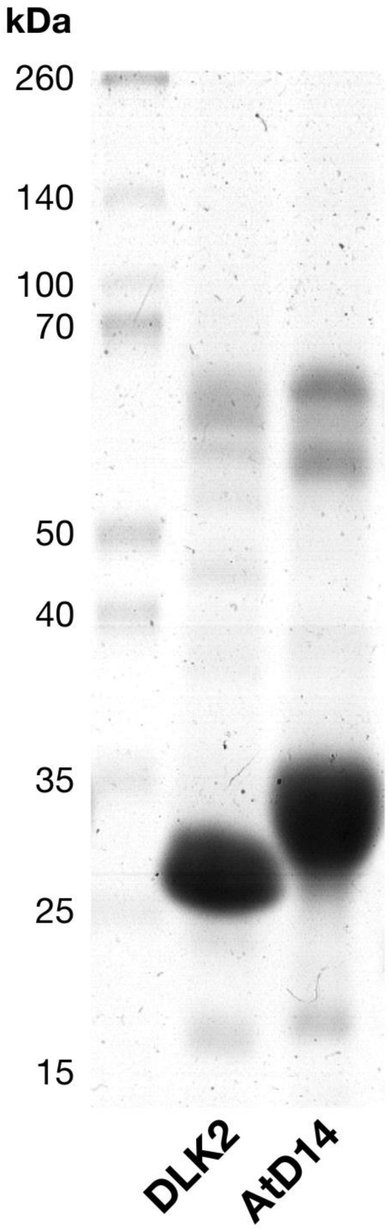

Supplemental Figure S1. SDS-PAGE of recombinant D14 and DLK2 His-tag phusion proteins. Proteins were purified on Ni-NTA columns as described in the Materials and Methods section. 20 µg of each protein was separated on 12% acrylamide gel, and stained with Coomassie BB. The predicted size of DLK2 and D14 His-tag phusion proteins were 31 and 32 kDa, respectively. Purity was estimated to be 90%.

Supplemental Figure S2.

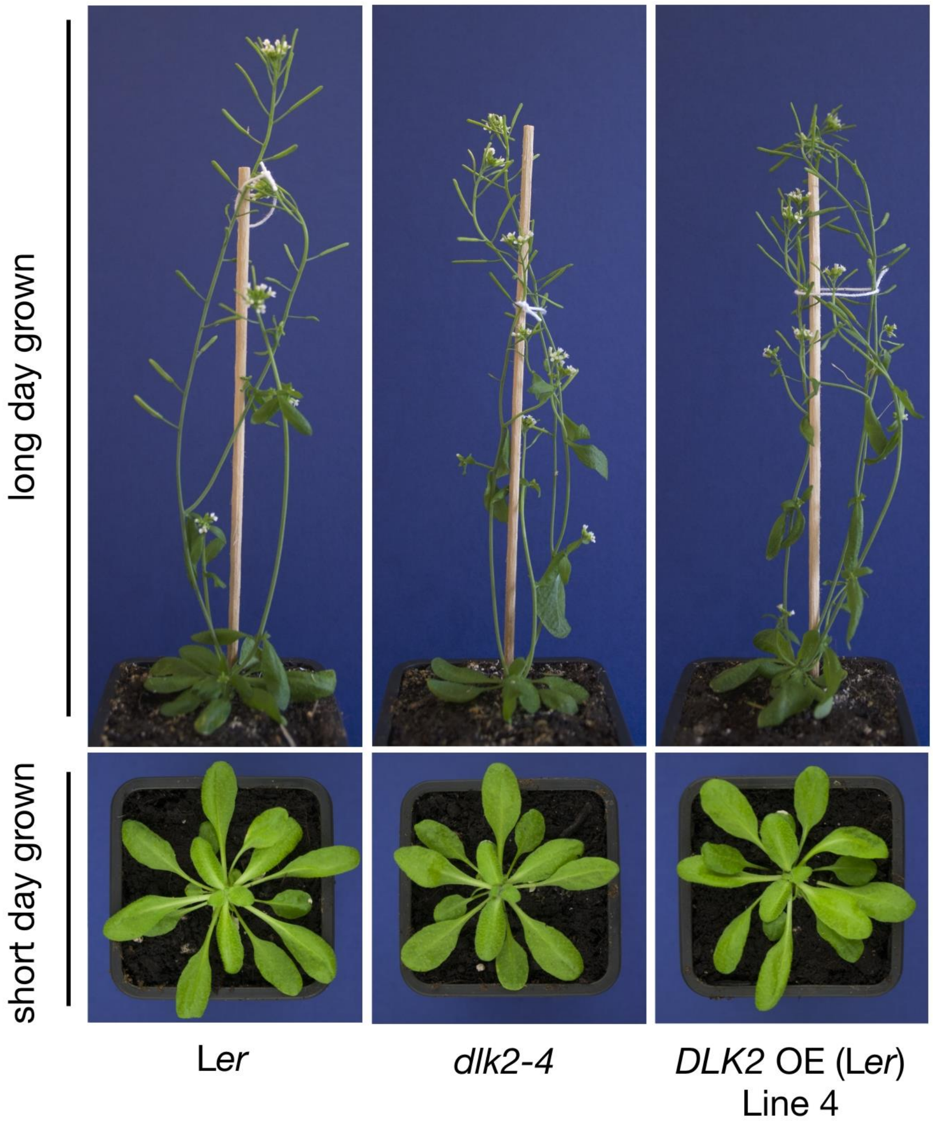

Supplemental Figure S2. The stature of *dlk2-4* and *DLK2* OE (L*er*) plants are similar to wild type L*er* plants. Plants were grown on either a long day (16h/8h day and night) or short day (8h/16h day and night) photoperiod (80 µmol m^{-2} s^{-1}; 21°C /18°C day and night temperature, 75% relative humidity) for 50 or 30 days, respectively.

Supplemental Figure S3.

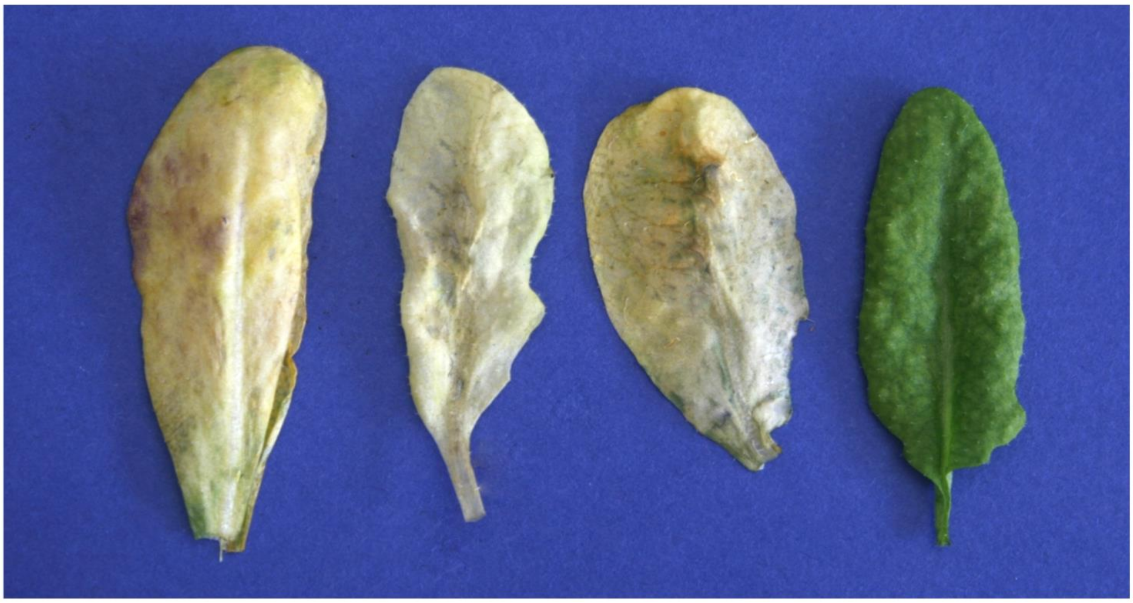

Supplemental Figure S3. Leaf senescence is not delayed in *dlk2-4* and *DLK2* OE (L*er*) plants. Senescence tests were accomplished using 6-week-old plants according to Ueda and Kusaba (2015).

Supplemental Figure S4.

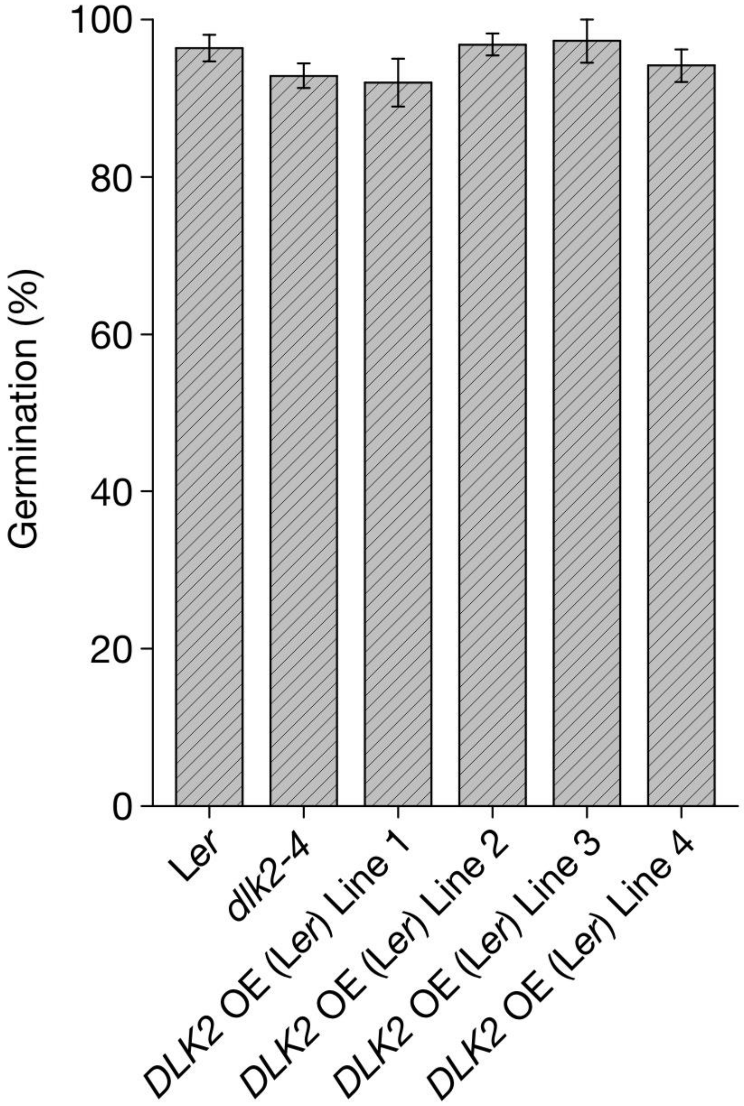

Supplemental Figure S4. *DLK2* OE (L*er*) and *dlk2-4* mutants exhibit normal germination. Siliques which just turned yellow were collected, immediately sterilized and sown on 0,5×MS plates with 1% sucrose. Germination was scored for 8 d. Germination data collected on day 6 are presented (three biological replicates, n=50 seeds in each; mean±SD).

Supplemental Figure S5.

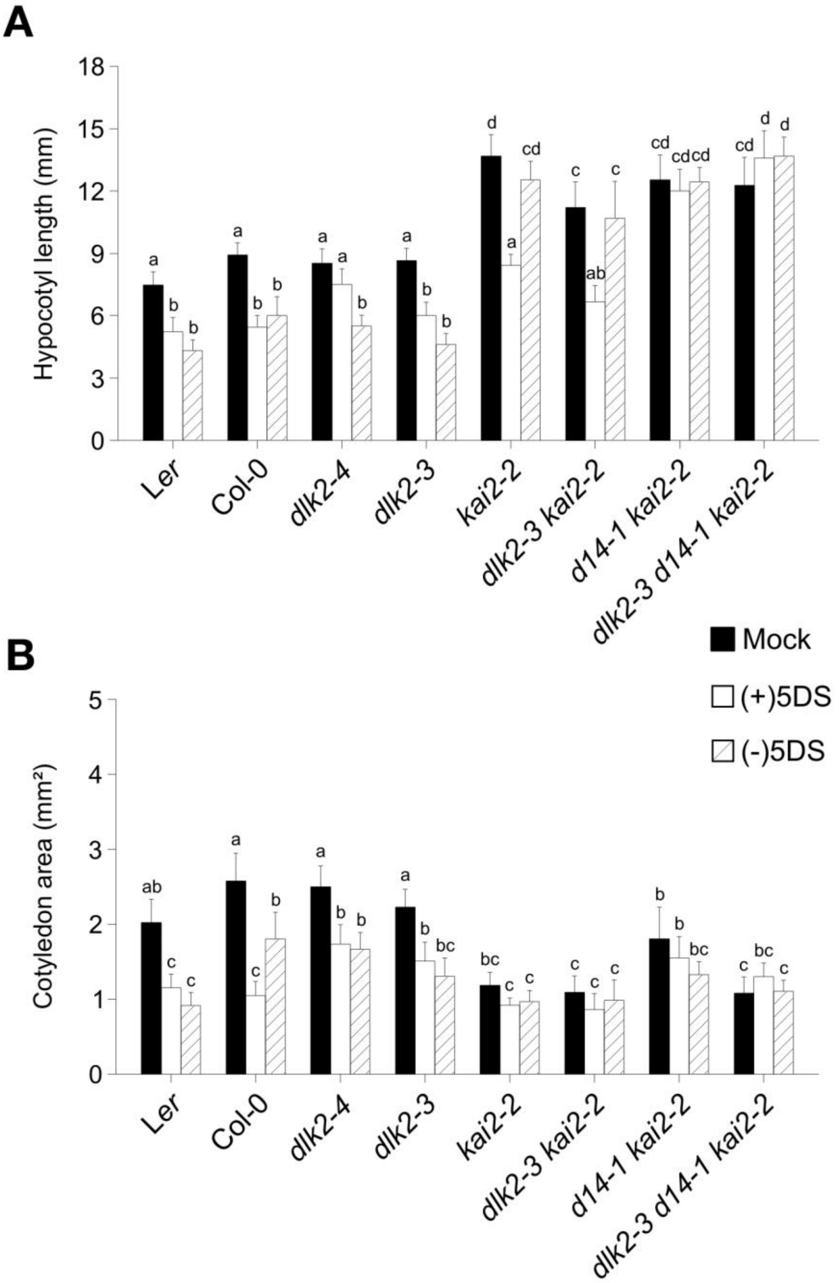

Supplemental Figure S5. A-B, Hypocotyl elongation (A) and cotyledon expansion (B) responses of low light grown (8 days) *dlk2-3*, *kai2-2* (in L*er* background) mutants and their combinations to 10 µM of (+)5DS and (-)5DS applications as compared to wild type L*er* and Col-0 seedlings. Data are means of 5 independent experiments, >30 seedlings in each. Bars with the same letter are not significantly different from each other (mean±SD; ANOVA, $P < 0.01$, Tukey's HSD test).
